# Phosphofructokinase Platelet Overexpression Accelerated Colorectal Cancer Cell Growth and Motility

**DOI:** 10.7150/jca.82738

**Published:** 2023-04-02

**Authors:** Tzung-Ju Lu, Yi-Fen Yang, Ching-Feng Cheng, Ya-Ting Tu, Yi-Ru Chen, Ming-Cheng Lee, Kuo-Wang Tsai

**Affiliations:** 1 Division of Colon and Rectal Surgery, Department of Surgery, Taipei Tzu Chi Hospital, Buddhist Tzu Chi Medical Foundation, New Taipei City, Taiwan; 2 Pulmonary function Laboratory, Division of Pulmonary Medicine, Kaohsiung Medical University Chung-Ho Memorial Hospital; 3 Department of Pediatrics, Taipei Tzu Chi Hospital, Buddhist Tzu Chi Medical Foundation, Taipei, Taiwan; 4 Institute of Biomedical Sciences, Academia Sinica, Taipei, Taiwan; 5 Department of Pediatrics, Tzu Chi University, Hualien, Taiwan; 6 Department of Research, Taipei Tzu Chi Hospital, Buddhist Tzu Chi Medical Foundation, New Taipei, Taiwan; 7 Department of Nursing, Cardinal Tien Junior College of Healthcare and Management, Taiwan

**Keywords:** phosphofructokinase platelet, colorectal cancer, metastasis

## Abstract

**Background:** Glycolysis is a glucose metabolism pathway that generates the high-energy compound adenosine triphosphate, which supports cancer cell growth. Phosphofructokinase platelet (PFKP) plays a crucial role in glycolysis regulation and is involved in human cancer progression. However, the biological function of PFKP remains unclear in colorectal cancer (CRC).

**Methods:** We analyzed the expression levels of PFKF in colon cancer cells and clinical samples using real-time PCR and western blot techniques. To determine the clinical significance of PFKP expression in colorectal cancer (CRC), we analyzed public databases. In addition, we conducted *in vitro* assays to investigate the effects of PFKP on cell growth, cell cycle, and motility.

**Results:** An analysis by the Cancer Genome Atlas database revealed that PFKP was significantly overexpressed in CRC. We examined the levels of PFKP mRNA and protein, revealing that PFKP expression was significantly increased in CRC. The results of the univariate Cox regression analysis showed that high PFKP expression was linked to worse disease-specific survival (DSS) and overall survival (OS) [DSS: crude hazard ratio (CHR) = 1.84, 95% confidence interval (CI): 1.01-3.36, *p* = 0.047; OS: CHR=1.91, 95% CI: 1.06-3.43, *p* = 0.031]. Multivariate Cox regression analysis revealed that high PFKP expression was an independent prognostic biomarker for the DSS and OS of patients with CRC (DSS: adjusted HR = 2.07, 95% CI: 1.13-3.79, *p* = 0.018; AHR = 2.34, 95% CI: 1.29-4.25, *p* = 0.005). PFKP knockdown reduced the proliferation, colony formation, and invasion of CRC cells. In addition, the knockdown induced cell cycle arrest at the G0/G1 phase by impairing cell cycle-related protein expression.

**Conclusion:** Overexpression of PFKP contributes to the growth and invasion of CRC by regulating cell cycle progression. PFKP expression can serve as a valuable molecular biomarker for cancer prognosis and a potential therapeutic target for treating CRC.

## Introduction

Colorectal cancer (CRC) is a highly common cancer globally and a main cause of cancer-related deaths 1. According to data from the United States, the diagnostic rate for CRC is 34 per 100,000 persons; in 2019, the cancer death rate was 12.8 per 100,000 persons 2. The 2021 Taiwan Cancer Registry Annual Report revealed that the incidence of CRC was 42.94 per 100,000 persons and that CRC was the second most common cancer. Therefore, the prediction, diagnosis, and treatment of CRC are crucial for CRC prevention and have been studied extensively through various methods.

During glycolysis, one molecule of glucose is converted into two molecules, one is pyruvate and the other is adenosine triphosphate (ATP). The tricarboxylic acid (TCA) cycle and the respiratory chain can oxidize pyruvate to CO2 and H2O in aerobic conditions, yielding large quantities of ATP 3. Therefore, glucose metabolism is the most crucial pathway that provides ATP to the human body; ATP is well known as energy currency. In normal human cells, ATP is generated through oxidative phosphorylation in mitochondria. In cancer cells, ATP is crucial because the demand is higher than in normal cells 4. Anaerobic glycolysis increases in cancer cells, a phenomenon first described by Otto Warburg in 1920s 5. In most cancer cells, pyruvate is converted into lactate in the cytoplasm rather than being oxidized through the TCA cycle. Although malignant cells have a sufficient amount of oxygen, they exhibit significantly increased anaerobic glycolytic activity; this is considered a fundamental metabolic reprogramming mechanism 6. Several mechanisms have been suggested to modulate the Warburg effect, including mitochondrial defects, a hypoxic environment, oncogenic signal activation, and metabolic enzyme deregulation 7-13.

One study revealed that tumor cells can increase growth, metastasis, and drug resistance through metabolic reprogramming 14. Another study also demonstrated the relationship between glucose metabolic enzymes and cancers, revealing that cancer cells are highly glycolytic and actively uptake glucose 15. Therefore, the expression levels of glycolysis-related enzymes, including glucose transporters 16-18, glucose-6-phosphate isomerase 19, glyceraldehyde 3-phosphate dehydrogenase 20, and hexokinase 21, are frequently abnormal during CRC progression. These studies have demonstrated that cancer cells exhibit increased glycolysis and are more dependent on this pathway for ATP generation.

Phosphofructokinase-1 (PFK-1) is a crucial enzyme in glycolysis that phosphorylates fructose 6-phosphate to fructose 1,6-bisphosphate. PFK-1 has three isoforms, PFKP, PFKM, and PFKL. PFKL is most abundant in the liver and kidneys, whereas PFKM and PFKP appear in muscles and platelets, respectively. Three isoforms of PFK-1 have been noted, with different proportions in different cancers 22. Among these isoforms, PFKP is frequently abnormally expressed in lung cancer, breast cancer, kidney cancer, and oral squamous cell carcinoma and has a poor effect on cancer behavior 23-26. However, the clinical effect and biological role of PFKP in colorectal cancer remain unclear. This study explored the putative role of PFKP in colorectal carcinogenesis.

## Materials and Methods

### Cell Line

Five CRC cell lines, SW620, LoVo, SW480, DLD-1, and HCT116, were obtained from the American Type Culture Collection and cultured in Dulbecco's modified Eagle's medium with 10% inactivated fetal bovine serum (Gibco, Thermo Fisher Scientific Inc., Waltham, MA, USA).

### Expression Data from Public Database

A total 465 RNA expression profiles were obtained from the TCGA data portal (https://tcga-data.nci.nih.gov/tcga/dataAccessMatrix.htm), including 41 corresponding adjacent normal tissues and 424 CRC. The clinical information of the patients with CRC was also obtained from TCGA, including gender, age, and pathological stage, as well as overall survival, disease-specific survival, and progress-free survival. We excluded patients who lacked this information from this version of the study. Furthermore, the normal tissues (breast, colon, lung and stomach) were downloaded from The Genotype-Tissue Expression databases (GTEx) (http://gtexportal.org/home/).

### Clinical Samples

Eighteen CRC tissues and corresponding adjacent normal mucosa samples were obtained from the Biobank of Taipei Tzu Chi Hospital, Taiwan. Informed consent was obtained from all patients by the Biobank of Taipei Tzu Chi Hospital. This study was approved by the ethics committee of Taipei Tzu Chi Hospital (09-X-008).

### Real-Time Reverse Transcription-PCR

Total RNA was extracted using the EasyPrep Total RNA Kit (Biotools Co., Ltd., Taipei, Taiwan). The concentration and purity of the total RNA were determined using the NanoDrop 1000 spectrophotometer (NanoDrop Technologies Inc., DE, USA). Subsequently, 2 µg of the total RNA was reverse-transcribed into cDNA by using oligo (dT) 15 primers and the ToolScript MMLV RT Kit in accordance with the instruction manual (Biotools Co., Ltd., Taipei, Taiwan). The expression of individual genes was quantified using SYBR-Green-I-based real-time reverse transcription-polymerase chain reaction (RT-PCR; Biotools Co., Ltd., Taipei, Taiwan) with gene-specific primers. The expression levels of S26 were used as an internal control. The sequence of PFKP primers was as follows:

PFKP-F: 5′- ATTTGTGTGCTGGGAATAAG -3′

PFKP-R: 5′- GGAATCCTGTGCTCAAAATC -3′

S26-F: 5′- CCGTGCCTCCAAGATGACAAAG -3′

S26-R: 5′- GTTCGGTCCTTGCGGGCTTCAC -3′

### Western Blotting

The cells were lysed with lysis buffer (50 mM Tris-HCl at pH 8.0, 150 mM NaCl, 1% NP-40, 0.02% sodium azide, 1 μg/mL aprotinin, and 1 mM PMSF) at 4 ℃ for 30 min. The concentration of individual cell lysate was assessed using the Bio-Rad DC Protein Assay Kit (500-0113, Bio-Rad Laboratories, Inc., Hercules, CA, USA). Protein samples were separated using a 10% sodium dodecyl sulfate-polyacrylamide gel. The separated proteins were electrotransferred onto a nitrocellulose membrane (Amersham Pharmacia Biotech). The expression levels of individual proteins were analyzed by incubating the membrane with corresponding primary antibodies in phosphate-buffered saline with Tween20 (PBST) and 5% skim milk at 4 ℃ overnight. Subsequently, the membrane was incubated with antirabbit or antimouse immunoglobulin G horseradish peroxidase-conjugated secondary antibody (1:10,000, Roche Molecular Biochemicals) for 1 h at room temperature. The membrane was washed three times with PBST, and the individual protein expression was detected using an electrochemiluminescence kit (Biotools Co., Ltd., Taipei, Taiwan). The following primary antibodies were used: CCNA2 (1:1000; 18202-1-AP, Proteintech Group, Inc., Rosemont, IL, USA), CCNB1 (1:1000; 55004-1-AP, Proteintech Group, Inc., Rosemont, IL, USA), CCND1 (1:1000; RM-9104-S, Thermo Fisher Scientific Inc., Waltham, MA, USA), CDK1 (1:200; 10762-1-AP, Proteintech Group, Inc., Rosemont, IL, USA), CDKN1A (1:1000; #2947, Cell Signaling Technology, Inc., Beverly, MA, USA), and ACTB (1:2000, MAB1501, EMD Millipore, Billerica, MA, USA).

### Cell Proliferation, Migration, and Invasion Analysis

To analyze cell proliferation, 5000 CRC cells were cultured in 96-well plates. Cell growth was determined at 0, 1, 3, and 5 days by using 3-(4,5-dimethylthiazol-2-yl)-2,5-diphenyltetrazolium bromide (MTT; Sigma-Aldrich, St. Louis, MO, USA). The migration and invasion of CRC cells were examined *in vitro* by using transwell chambers (Corning Life Sciences, Lowell, MA, USA). CRC cells with PFKP knockdown were seeded in the upper chamber (containing 8-µm pores) of a transwell and incubated for 24 h at 37 ºC. Subsequently, the cells in the lower chamber were stained with Giemsa stain. Cell migration or invasion was determined using a microscope at 200× magnification. All experiments were repeated three times.

### Cell Cycle and Apoptosis Analysis

CRC cells with or without PFKP knockdown were collected and washed with PBS. Subsequently, the cells were stained with 50 mL of propidium iodide (Boehringer Mannheim Corp; Roche Molecular Biochemicals, Basel, Switzerland). Filtration was performed to remove cell aggregates, and the cell cycle was analyzed using a Coulter Epics XL Flow Cytometer.

### Glucose Uptake Assay

To analyze cellular glucose uptake, HCT116 cells with PFKP knockdown were cultured in a glucose-free medium. Subsequently, 200 µg/mL of 2-NBDG, a fluorescently labeled compound (ab235976, Abcam), was added to the cells and cultured for 48 h. The fluorescent signals were detected using fluorescence microscopy.

### Statistical Analysis

Data on PFKP expression levels in CRC tissues from the TCGA database and GTEx database were analyzed using a Student's *t* test. The PFKP expression levels obtained using real-time RT-PCR were examined using a paired *t* test. The chi-square test, Student's *t* test, analysis of variance (ANOVA), Mann-Whitney U test, and Kruskal-Wallis one-way ANOVA were used to evaluate the correlation between PFKP expression levels and clinicopathological parameters of patients with CRC. To analyze the overall survival, disease-specific survival and progress-free survival, the patients were divided into high- and low-PFKP expression groups by using the receiver operating characteristics curve to define the best cutoff value. The cumulative survival curves of the patients with CRC were evaluated using the Kaplan-Meier method. The overall survival curve of the patients in both groups was assessed using the log-rank test. All biological functional assays were performed in triplicate. Student's *t* tests were used to analyze these data, and *p* < 0.05 indicated a significant difference between the groups.

## Results

**Figure 1:**
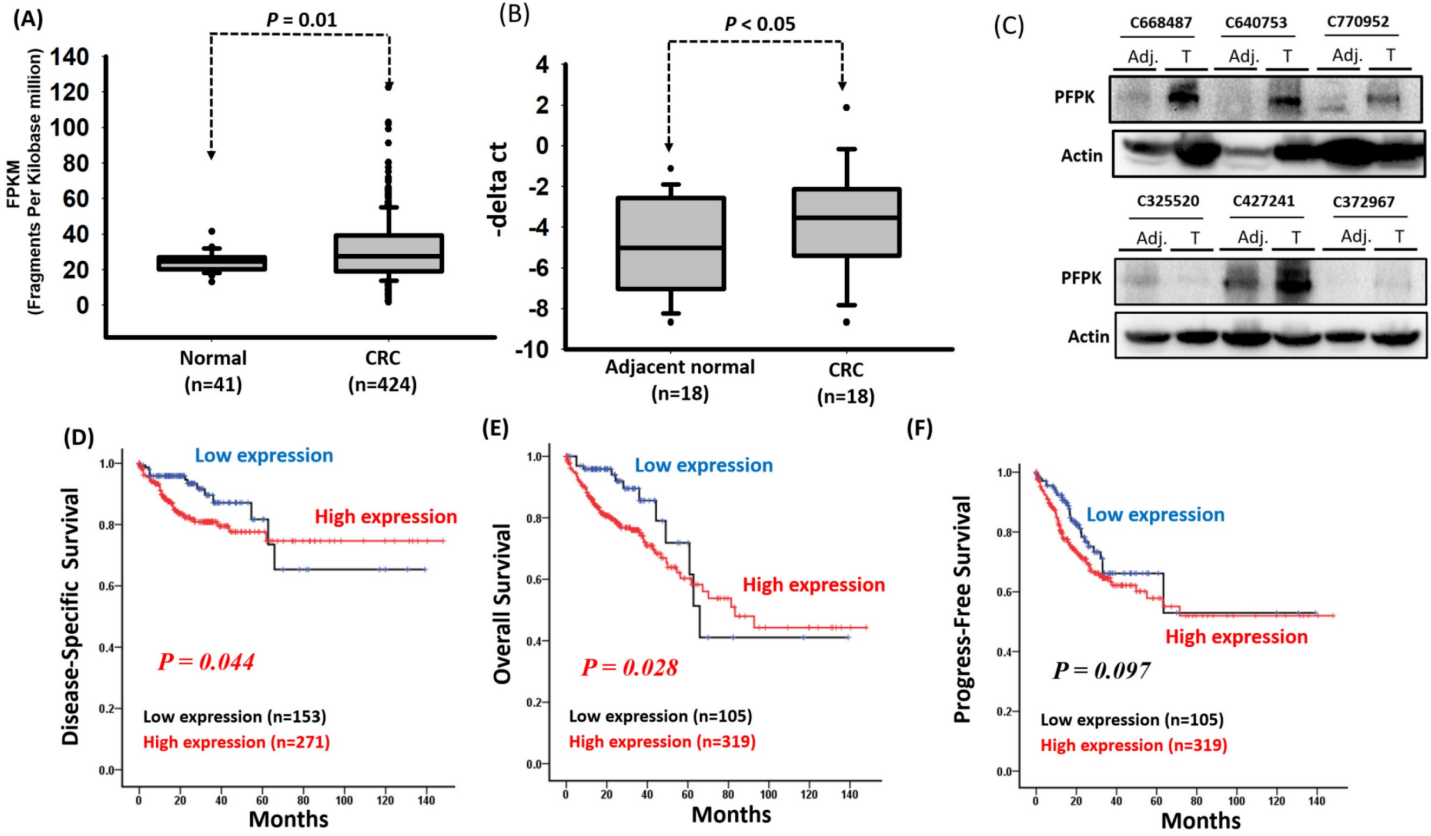
** PFKP overexpression in human colorectal cancer.** (A) PFKP expression was examined in human colorectal cancer by using the TCGA database. (B) PFKP expression was examined in 18 N-T paired human colorectal cancer tissues by using real-time RT-PCR. (C) Western blotting for PFKP protein extracts in colorectal cancer. (D), (E) and (F) Correlation between PFKP expression and disease-specific survival (DSS), overall survival (OS) and progress-free survival (PFS) of patients were assessed using the Kaplan-Meier method.

Previous studies have reported that PFKP is overexpressed in several human cancers 25, 26, 27. To confirm these findings, we downloaded the expression levels of PFKP from the GTEx database (for human normal tissues) and TCGA database (for human tumor tissues). As shown in Supplementary Fig. 1, the expression levels of PFKP were significantly increased in tumor tissues compared to normal tissues, including breast, colon, lung, and stomach. Among these, the expression levels of PFKP were significantly increased in corresponding adjacent normal (p=5.78e^-05^) and colorectal carcinoma tissue (p<2.2e^-16^) compared to colon normal tissues (Supplementary Fig. 1). However, the impact of PFKP expression on the progression of colorectal cancer (CRC) in patients remains unknown. Therefore, we downloaded RNA transcriptome data and detailed clinical information of patients with CRC from the Cancer Genome Atlas (TCGA) database, which included 41 corresponding adjacent normal tissues and 424 colon cancer tissues. The clinical and pathological information of the patients is summarized in Supplementary Table 1. Our results indicate that the PFKP expression levels were significantly higher in the CRC tissues than in the corresponding adjacent normal tissues (*p* = 0.01; Fig. 1A). To confirm the expression of PFKP, we examined the mRNA and protein levels in eighteen CRC tissues and corresponding adjacent normal tissues by real-time polymerase chain reaction (PCR) and western blot approach. Our data revealed that the higher PFKP expression levels in the CRC tissues than in the adjacent normal tissues (Fig. 1B and C). Further examination of the clinical effects of PFPK on CRC revealed that PFPK expression was nonsignificantly correlated with clinicopathological features (Table 1). Kaplan-Meier analysis revealed that high PFKP expression was significantly correlated with a worse disease-specific survival (DSS) and overall survival (OS) curve for patients with CRC (DSS: *p* = 0.044 and OS: *p* = 0.028), and breadline significant associated disease progression (*p* = 0.097) (Fig. 1D-1F). The multivariate Cox regression analysis indicated that PFKP expression was associated with poor DSS [adjusted hazard ratio (AHR): 2.07; 95% confidence interval (CI): 1.13-3.79; *p* = 0.018] and OS (AHR: 2.34; 95% CI: 1.29-4.25; *p* = 0.005) in patients with CRC (Table 2-4).

### Involvement of PFKP in CRC Cell Motility and Growth

**Figure 2:**
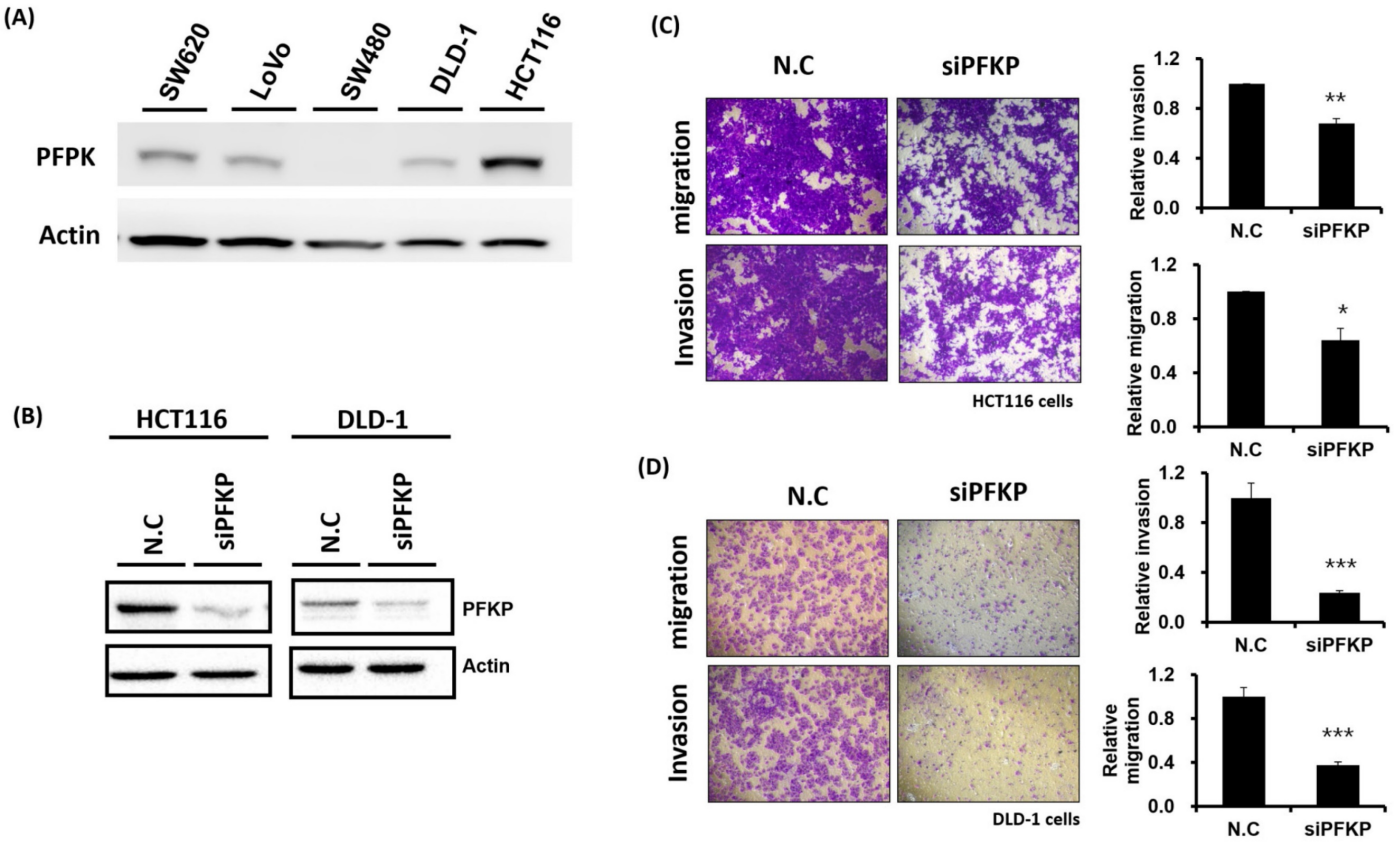
** Suppression of colorectal cancer cell motility by PFKP knockdown.** (A) PFKP expression levels in five colon cancer cell lines (SW620, LoVo, SW480, DLD-1, and HCT116) were examined using real-time RT-PCR. (B) siRNA-mediated suppression of PFKP expression in HCT116 and DLD-1 cells. After transfection of the cells with siPFKP for 48 h, PFKP expression levels were examined using Western blotting. (C) and (D) Invasion and migration of HCT116 and DLD-1 cells were assessed using transwell assays. After siPFKP transfection for 48 h, the cells were subjected to transwell assays and incubated at 37 °C for 48 h. Invasion and migration of the transfected cells were quantified.

**Figure 3:**
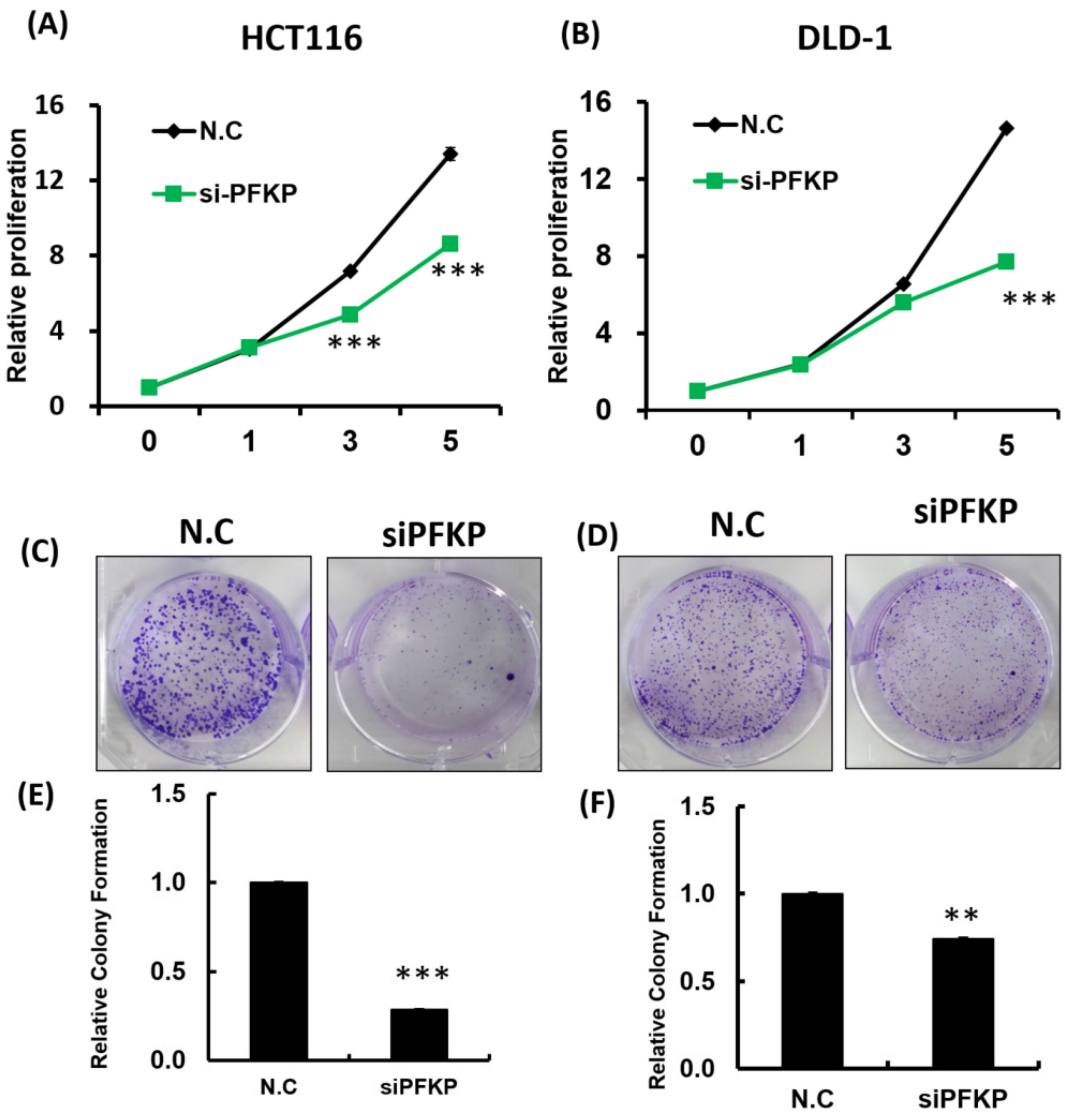
** PFKP knockdown influenced colorectal cancer cell growth.** (A) and (B) HCT116 and DLD-1 cells were seeded in 96-well plates and transfected with siPFKP; cell proliferation was measured at 0, 1, 3 and 5 days. Colony formation of HCT116 and DLD-1 cells was examined after siPFKP transfection for 1 week. Images from a representative experiment are displayed (C and D) together with data quantification (E and F).

**Figure 4:**
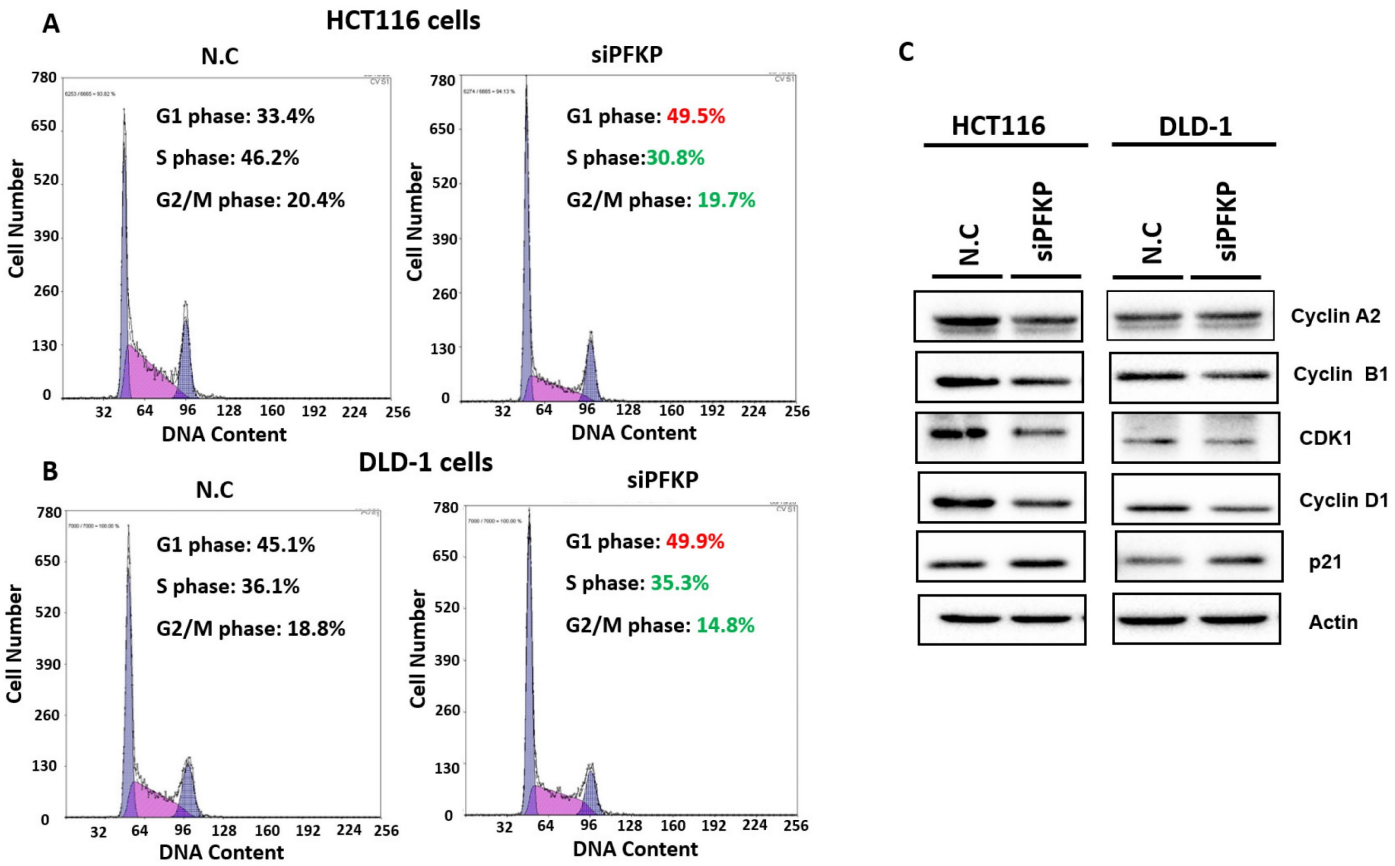
** PFKP knockdown impaired cell progression by silencing cell cycle-related gene expression.** (A) and (B) After siPFKP transfection for 48 h, the cell cycle was examined in HCT116 and DLD-1 cells by using flow cytometry. (C) Expression levels of cell cycle-related genes were examined in HCT116 and DLD-1 cells with PFKP knockdown by using Western blotting.

Our above results indicate that PFKP expression was overexpressed in CRC. To explore the biological function of PFKP in CRC, we used the loss-of-function approach of siRNA knockdown. First, we examined PFKP expression in CRC cell lines by using Western blotting. PFKP expression was high in HCT116 cells; medium in DLD-1, SW620, and LoVo cells; and low in SW480 cells (Fig. 2A). We selected HCT116 and DLD-1 cells with high PFKP expression for siRNA transfection. The results indicate that PFKP expression decreased in cells transfected with siPFKP for 48 h (Fig. 2B). Subsequently, the functional assays, comprising glucose uptake, cell migration, cell invasion, colony formation, and cell proliferation assays, were performed. The results indicate that PFKP knockdown decreased glucose uptake in HCT116 cells (Supplementary Fig. 2). The transwell assay revealed that PFKP knockdown significantly decreased CRC cell migration and invasion (Fig. 2C and D). We also examined the growth of HCT116 and DLD-1 cells after PFKP knockdown. Our results indicate that PFKP knockdown significantly suppressed proliferation and colony formation in HCT116 and DLD-1 cells (Fig. 3A-3F). We also analyzed cell cycle and cell apoptosis in HCT116 cells after PFKP knockdown. The knockdown induced cell arrest at the G1 phase and reduced the population of HCT116 and DLD-1 cells in the S and G2/M phases (Fig. 4A and 4B). We examined the expression levels of cell cycle-related genes, and the results indicate decreased expression of cyclin A2, B1, D1, and CDK1 and increased expression of p21 (Fig. 4C). However, PFKP knockdown did not influence CRC cell apoptosis (Supplementary Fig. 3). Therefore, PFKP knockdown may suppress CRC cell growth by inducing cell cycle arrest at the G1 phase. Taken together, our data revealed that PFKP overexpression may accelerate CRC cell growth and metastasis. Furthermore, high PFKP expression may be a poor prognostic biomarker for the overall survival of patients with CRC.

## Discussion

PFKP is a crucial enzyme in glycolysis that phosphorylates fructose 6-phosphate to fructose 1,6-bisphosphate. Studies have revealed that PFKP has been overexpressed in human cancers, including lung cancer, oral cancer, hepatocellular carcinoma, breast cancer, and clear cell renal cell carcinoma 27-32 Some studies have reported that high PFKP expression was correlated with poor prognosis, lymph node metastasis, histological grade, T category, and survival in patients with lung cancer 30, 33. Herein, our data showed that PFKP was significantly unregulated in CRC, whereas no correlation with pathological features.

Except for CRC, the biological function of PFKP was be clear examined in human cancer. In lung cancer cells, PFKP knockdown significantly decreased the glucose uptake rate and ATP production 30. One study silenced PFKP expression causing cell cycle arrest at the G2/M phase, which significantly suppressed colony formation and proliferation of lung cancer cells 30.

Wang et al. reported that PFKP overexpression inhibited cancer cell apoptosis and accelerated the growth, migration, and invasion of H1299 cells 33. In breast cancer, PFKP expression was negatively correlated with estrogen receptor and human epidermal growth factor receptor-2 and positively correlated with transforming growth factor-β and MYC expression 34. A study reported that PFKP expression was positively associated with estrogen receptor- and progesterone receptor-negative breast cancers 29. Decreased PFKP expression significantly suppressed breast cancer cell growth and invasion 34. A dietary polyphenolic compound, quercetin, suppressed growth and migration by inhibiting PFKP and LDHA expression in breast cancer 29. Wang et al. also reported that the suppression of PFKP expression in clear cell renal cell carcinoma inhibited cancer cell growth by impairing cell cycle progression and inducing apoptosis 35. Chen et al. reported that starvation induced PFKP overexpression and that PFKP knockdown significantly suppressed starvation-mediated autophagy, growth, and invasion in oral squamous cell carcinoma 31. PFKP knockdown together with metabolic reprogramming suppressed glycolysis, the pentose phosphate pathway, and nucleotide biosynthesis in human cancer; however, it increased TCA cycle activity 29, 31, 32, 35. Our study is the first to report that PFKP overexpression is significantly associated with poor survival in patients with CRC. As observed in other cancers, the inhibition of PFKP expression decreased CRC cell growth and motility and impaired cell cycle progression (Fig. 2-4). In addition, PFKP knockdown suppressed glucose uptake in CRC. However, our results revealed that PFKP knockdown could not induce colon cancer apoptosis (Supplementary Fig. 2). Different from previous research results, we found that PFKP knockdown can induce colon cancer cells arrest at G1 phase. These inconsistent results may be due to different types of cancer.

The unclear mechanism of PFKP-mediated cancer growth and metastasis is a weakness of our study. A study indicated that high PFKP expression induced intracellular citrate accumulation in breast cancer, leading to significantly enhanced cell migration and invasion through the AKT/ERK/MMP2/MMP9 signaling axis 36. Lee et al. reported that epidermal growth factor receptor (EGFR) activation accelerated the phosphorylation of PFKP Y64, which plays a critical role in human cancer cell growth and metastasis by modulating AKT activation 37. In addition, AKT phosphorylates PFKP at S386, resulting in PFKP protein stabilization by abrogating the binding of TRIM21 E3 ligase to PFKP 25. Jeon et al. reported that Wnt3A activated PI3K/AKT signaling in a β-catenin-independent manner. PI3K/AKT signaling activation accelerated PFKP S386 phosphorylation, resulting in PFKP upregulation through PFKP protein stabilization 38. Several transcription factors, including KLF-4, HIF-1α, and ZEB1, upregulated PFKP expression by directly binding to its promoter 36, 39, 40. In addition, some transcription factors, such as ZBTB7A, BRAC1/ZBRK1, and snail, downregulated PFKP expression 41-43. PFKP is predominantly expressed in the cytoplasm and plays a crucial role in glycolysis. Gao et al. indicated that the PFPK protein contains a nuclear localization sequence, which facilitates its shuttling between the nucleus and cytoplasm 44. High cyclin D3/CDK6 expression accelerated PFKP translocation into the nucleus by inducing PFKP and importin-9 interaction 44. Lee et al. indicated that EGFR activation resulted in K395 acetylation and PFKP Y64 phosphorylation. The phosphorylated PFKP recruited p85α to the plasma membrane and promoted PI3K/AKT signaling activation 45. However, the detailed mechanism of PFKP-mediated CRC growth and metastasis should be elucidated in subsequent studies.

In summary, PFKP overexpression contributes to CRC growth and invasion and PFKP expression can serve as a useful molecular biomarker for cancer prognosis and a potential therapeutic target for CRC.

## Supplementary Material

Supplementary figures and table.

## Figures and Tables

**Table 1:** Correlation of PFKP expression with clinicopathological characteristics of 424 patients with colon cancer.

Variables	PFKP (n=424)			
	No. (%)	Mean±SD	Median	p-value
**AJCC Pathology stage**				
I	74 (17.4)	30.34±16.05	28.11	0.349a
II	168 (39.6)	32.64±19.23	28.01	
III	122 (28.8)	31.07±15.82	27.78	
IV	60 (14.2)	28.12±14.99	25.87	
**T Classification**				
T1	10 (2.4)	35.00±11.14	30.94	0.630a
T2	73 (17.2)	29.05±15.95	27.26	
T3	288 (67.9)	31.44±17.85	27.44	
T4	53 (12.5)	31.77±16.20	30.77	
**N Classification**				
N0	251 (59.2)	31.83±18.24	27.95	0.289b
N1	98 (23.1)	28.26±12.86	26.38	
N2	75 (17.7)	32.67±18.27	30.33	
**M Classification**				
M0	364 (85.8)	31.65±17.50	27.95	0.141c
M1	60 (14.2)	28.12±14.99	25.87	
**Sex**				
Female	202 (47.6)	31.79±18.46	27.99	0.466^c^
Male	222 (52.4)	30.57±15.98	27.36	
**Age, years**				
<40	12 (2.8)	30.79±14.11	31.40	0.044^b^
40-59	105 (24,8)	27.51±14.09^d^	25.77	
≧60	307 (72.4)	32.41±18.11^d^	28.06	

^a^p-value were estimated by one-way ANOVA test. ^b^p-values were estimated by Kruskal-Wallis 1-way ANOVA test. ^c^p-value were estimated by Student's T test. ^d^p=0.014

**Table 2:** Univariate and multivariate Cox's regression analysis of PFKP expression for disease-specific survival of 424 patients with colon cancer.

Characteristic	No. (%)	DSS			
		CHR (95% CI)	P-value	AHR (95% CI)	P-value
**PFKP**	(n=424)				
**Low**	153 (36.1)	1.00		1.00	
**High**	271 (63.9)	1.84 (1.01-3.36)	**0.047**	2.07 (1.13-3.79)	0.018

^a^p-value were estimated by one-way ANOVA test. ^b^p-values were estimated by Kruskal-Wallis 1-way ANOVA test. ^c^p-value were estimated by Student's T test. ^d^p=0.014

**Table 3:** Univariate and multivariate Cox's regression analysis of PFKP gene expression for overall survival of 424 patients with colon cancer.

Characteristic	No. (%)	OS			
		CHR (95% CI)	P-value	AHR (95% CI)	P-value
**PFKP**	(n=424)				
**Low**	105 (24.8)	1.00		1.00	
**High**	319 (75.2)	1.91 (1.06-3.43)	**0.031**	2.34 (1.29-4.25)	**0.005**

Abbreviation: OS, Overall survival; CHR, crude hazard ratio; AHR, adjusted hazard ratio. AHR were adjusted for AJCC pathological stage (II, III and IV VS. I).

**Table 4:** Univariate and multivariate Cox's regression analysis of PFKP expression for progress-free survival of 424 patients with colon cancer.

Characteristic	No. (%)	PFS			
		CHR (95% CI)	P-value	AHR (95% CI)	P-value
**PFKP**	(n=424)				
Low	142 (33.5)	1.00		45.11.00	
High	282 (66.5)	1.42 (0.94-2.16)	0.099	1.50 (0.99-2.29)	0.058

Abbreviation: PFS, progress-free survival; CHR, crude hazard ratio; AHR, adjusted hazard ratio. AHR were adjusted for AJCC pathological stage (II, III and IV VS. I).
