# Supplementary material for: Phosphofructokinase Platelet Overexpression Accelerated Colorectal Cancer Cell Growth and Motility
Source: J Cancer. 2023 Apr 2;14(6):943–51. doi: 10.7150/jca.82738 (PMC10158518; doi:10.7150/jca.82738)
Supplement: Supplementary file 1 — Supplementary figures and table. [file jcav14p0943s1.pdf]

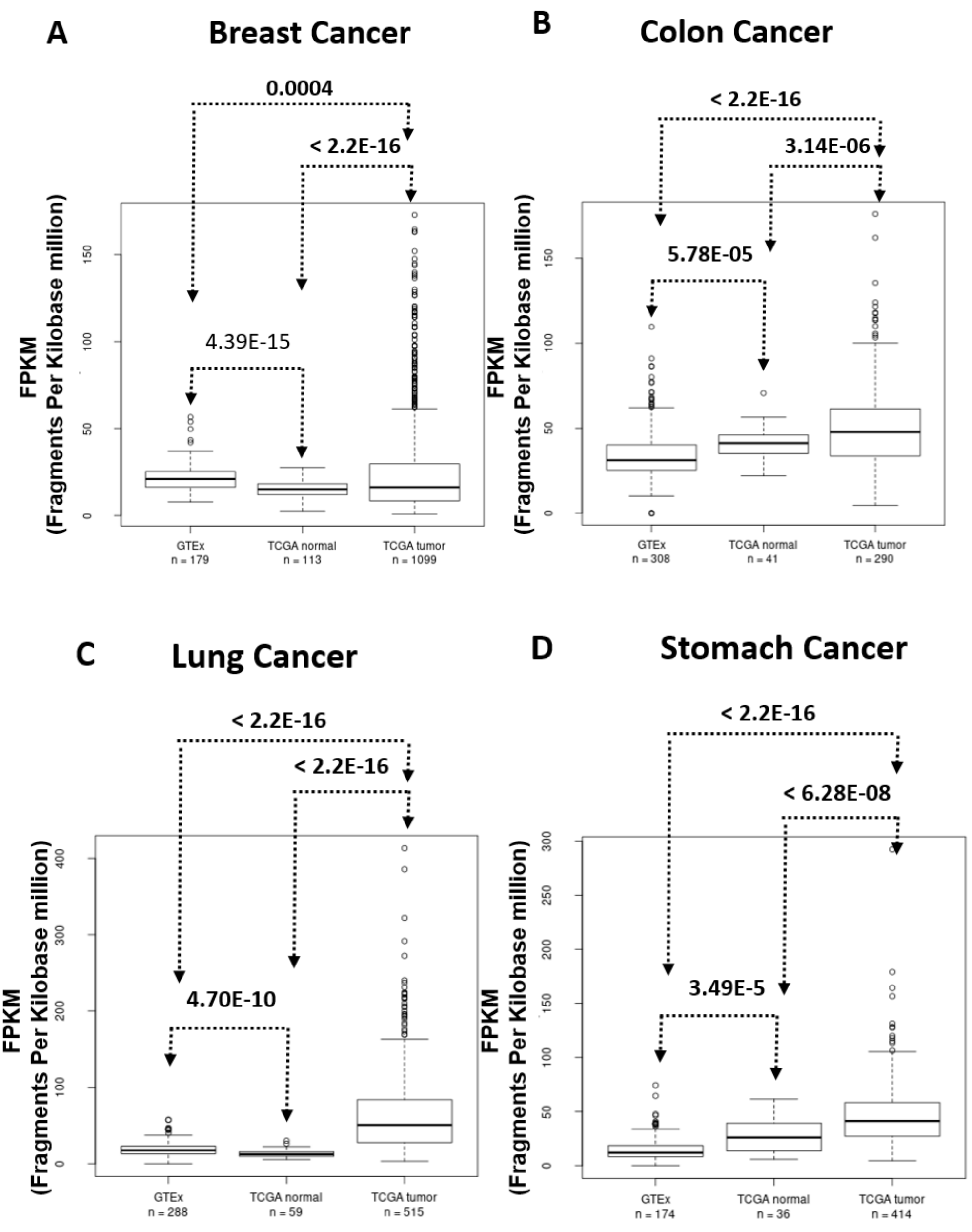

Supplementary Figure 1. The expression levels of PFKP were significantly increased in human cancer compared to corresponding normal tissues. The expression of PFKP in human normal tissues from The Genotype-Tissue Expression databases (GTEx) (http://gtexportal.org/home/), including colon, breast, lung, and stomach tissues. Combining data from GTEx and TCGA databases revealed that the expression levels of PFKP were significantly increased in breast cancer (A), colon cancer (B), lung cancer (C) and stomach cancer (D) compared to corresponding normal tissues.

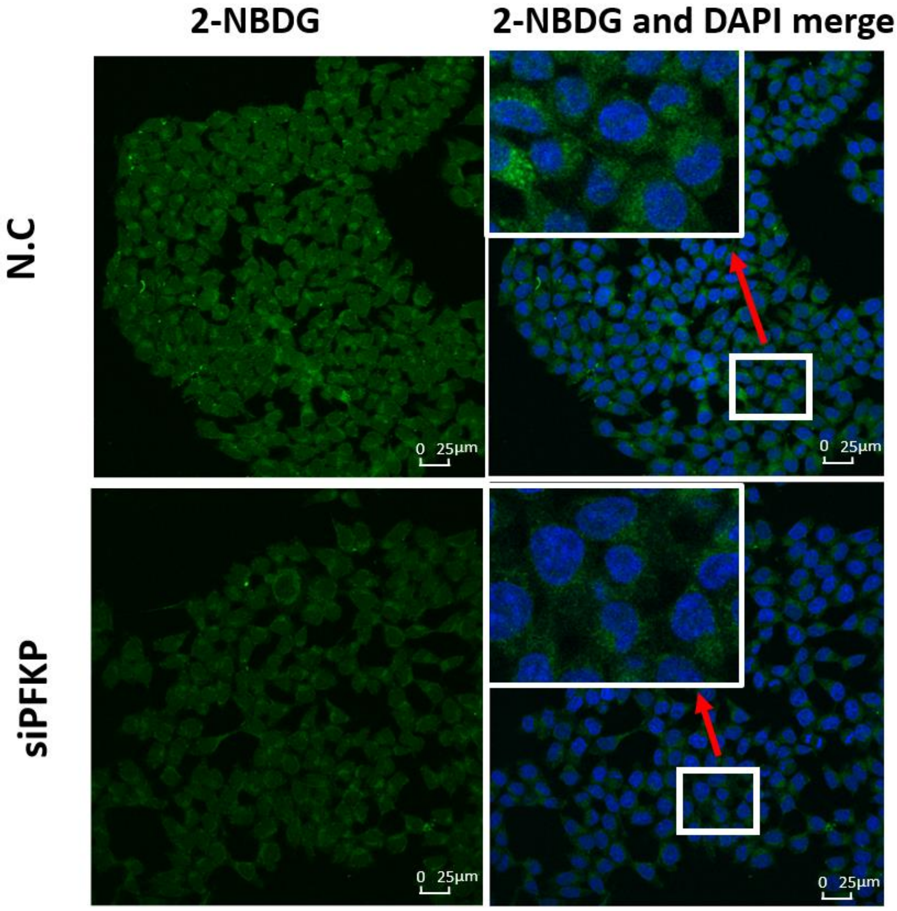

Supplementary Figure 2. PFKP knockdown decreased glucose uptake in colon cancer. The efficiency of glucose uptake was examined in HCT116 with PHKP knockdown using glucose uptake assay kit.

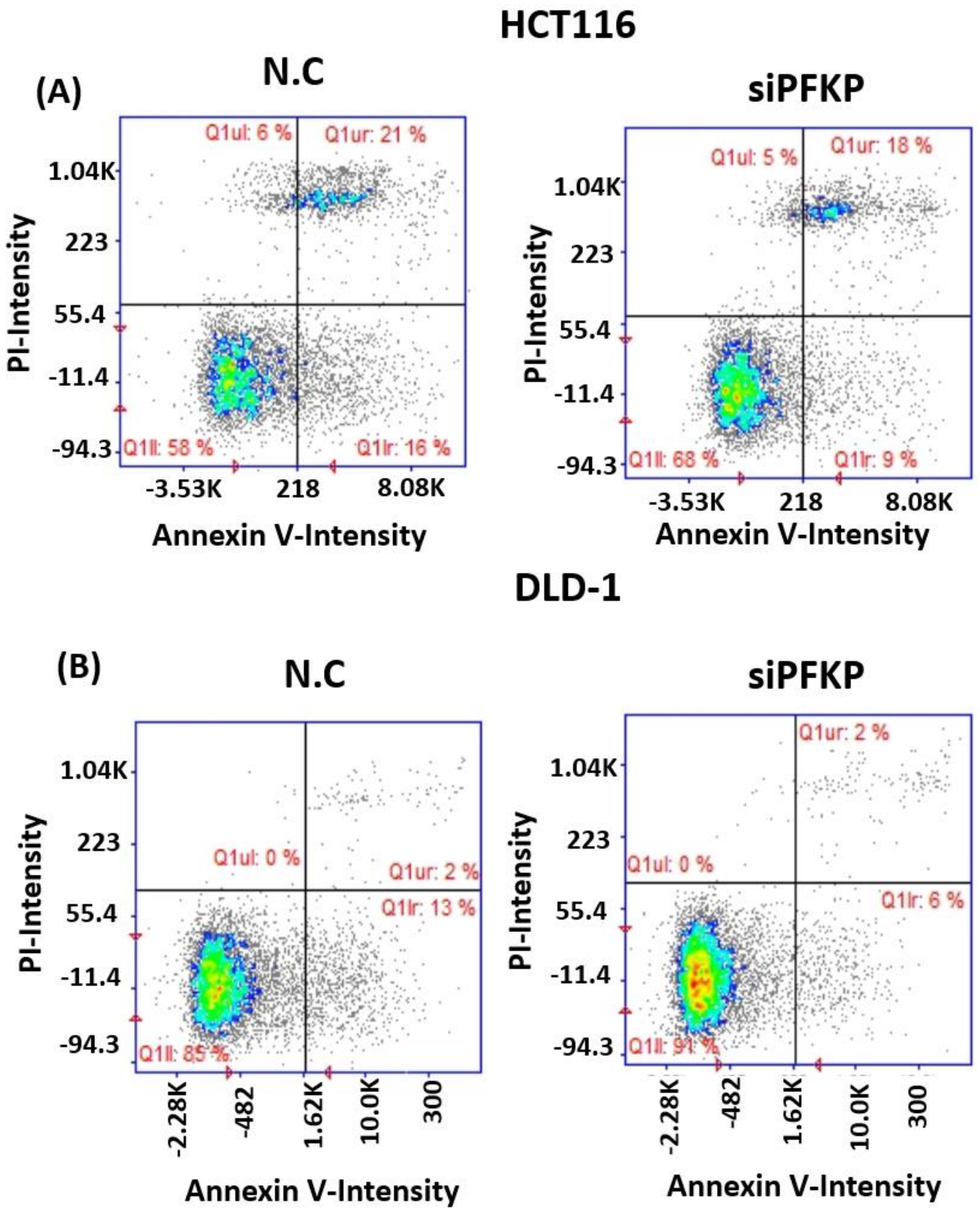

Supplementary Figure 3. PFKP knockdown did not influence HCT116 cell apoptosis. (A) and (B) HCT116 and DLD-1 cells were transfected with siPFKP for 48 h and triple stained with propidium iodide, annexin V, and Hoechst 33342 to analyze cell apoptosis through flow cytometry.

Supplementary Table 1: Demographic and clinicopathological characteristics of patients with CRC.

Variables	Numbers of patient	%
**Sex**		
Female	202	47.6
Male	222	52.4
**Age, years**		
<40	12	2.8
40-59	105	24.8
≧60	307	72.4
**T classification**		
T1	10	2.4
T2	73	17.2
T3	288	67.9
T4	53	12.5
**N classification**		
N0	251	59.2
N1	98	23.1
N2	75	17.7
**M classification**		
M0	364	85.8
M1	60	14.2
**AJCC Pathology stage**		
I	74	17.4
II	168	39.6
III	122	28.8
IV	60	14.2
